# Colchicine to Weather the Cytokine Storm in Hospitalized Patients with COVID-19

**DOI:** 10.3390/jcm9092961

**Published:** 2020-09-14

**Authors:** Luigi Brunetti, Oumou Diawara, Andrew Tsai, Bonnie L. Firestein, Ronald G. Nahass, George Poiani, Naomi Schlesinger

**Affiliations:** 1 Robert Wood Johnson University Hospital Somerset, 110 Rehill Avenue, Somerville, NJ 08876, USA; oumoudiawara2018@gmail.com (O.D.); andrew.tsai@rwjbh.org (A.T.); rnahass@idcare.com (R.G.N.); georgepoiani@hotmail.com (G.P.); 2 Ernest Mario School of Pharmacy, 160 Frelinghuysen Road, Piscataway, NJ 08854, USA; 3 Center of Excellence in Pharmaceutical Translational Research and Education, 160 Frelinghuysen Road, Piscataway, NJ 08854, USA; 4 Department of Cell Biology and Neuroscience, Rutgers, The State University of New Jersey, Piscataway, NJ 08854, USA; firestein@dls.rutgers.edu; 5 IDCare, Hillsborough, NJ 08844, USA; 6 Division of Pulmonary/Critical Care Medicine, Rutgers-Robert Wood Johnson Medical School, New Brunswick, NJ 08901, USA; 7 Division of Rheumatology, Department of Medicine, Rutgers Robert Wood Johnson Medical School, New Brunswick, NJ 08901, USA; schlesna@rwjms.rutgers.edu

**Keywords:** colchicine, COVID-19, viral pneumonia, coronavirus, cytokine storm

## Abstract

The repurposing of colchicine for the treatment of COVID-19 was suggested based in its immunomodulatory, anti-inflammatory, and anti-viral properties. We performed a single-center propensity score matched cohort study, including all consecutive COVID-19 patients admitted to a community hospital between 1 March 2020 and 30 May 2020. Patients were stratified according to the receipt of colchicine. The primary endpoint was defined as in-hospital death within 28-days follow-up. Secondary endpoints included favorable change in the Ordinal Scale for Clinical Improvement on days 14 and 28 versus baseline, proportion of patients not requiring supplemental oxygen on days 14 and 28, and proportion of patients discharged by day 28. In total data for 303 PCR positive COVID-19 patients were extracted and 66 patients were included in the 1:1 matched cohort study. At the end of the 28 day follow-up, patients receiving colchicine were approximately five times more likely to be discharged (odds ratio, 5.0; 95% confidence interval, 1.25–20.1; *p* = 0.023) and when comparing mortality, there were 3 deaths (9.1%) in patients receiving colchicine versus 11 deaths (33.3%) in the groups receiving standard of care (odds ratio, 0.20; 95% confidence interval, 0.05–0.80; *p* = 0.023). These observations warrant further investigation in large controlled clinical trials.

## 1. Introduction

The novel coronavirus disease 2019 (COVID-19), caused by severe acute respiratory syndrome–coronavirus-2 (SARS-CoV-2) infection, is a pandemic causing unprecedented medical, social, and economic havoc. Currently, off-label drug use is common in the treatment of COVID-19, for which limited therapies are available. Although antiviral drugs are expected to be a primary treatment modality, the use of immunomodulation and anti-inflammatories in these patients assumed a larger role particularly since a large study suggested the value of dexamethasone [1]. Reports on other anti-inflammatory medications produced mixed results.

Currently, there are no Food and Drug Administration (FDA) approved anti-inflammatory drugs to treat COVID-19 infection. Currently, there are insufficient clinical data to recommend either for or against the use of chloroquine, hydroxychloroquine, Interleukin-6 inhibitors (e.g., sarilumab, siltuximab, tocilizumab), and Interleukin-1 inhibitors (e.g., anakinra), except in the context of a clinical trial [2,3].

Colchicine, one of the oldest known drugs, has been used for over 2000 years ago in the form of preparations of the meadow saffron colchicum autumnale as a remedy for acute gout flares. Colchicine for oral use (capsule/tablet/liquid) is currently FDA approved for prevention and treatment of gout flares in adults and treatment of familial Mediterranean fever (FMF) [4]. Off-label uses for colchicine are multiple and include calcium pyrophosphate deposition disease, Behcet’s disease, amyloidosis, primary biliary cirrhosis, and pericarditis. Previous studies provide evidence that cardiovascular events are common in patients with severe COVID-19 [5]. Recent colchicine studies showing its efficacy in preventing major cardiovascular adverse events among patients who suffered a recent myocardial infarction compared with placebo is, therefore, of importance [6].

Colchicine has anti-inflammatory and anti-viral properties. The mechanism of action for potential benefit in severe COVID-19 is likely related to an inhibitory effect on the activation, destabilization, and degradation of inflammasomes, which may attenuate cytokine storm and has been recently described in detail [7]. Moreover, colchicine has antiviral properties mediated through microtubule polymerization inhibition [8,9,10]. Microtubules are involved in intracellular transport of viral particles [8,9]. Currently, ten colchicine clinical trials are in progress for the treatment of SARS-CoV-2 infection and are listed in clinicaltrials.gov [11]. Herein, we reported a cohort study in which we provided colchicine to patients with confirmed SARS-CoV-2 infection admitted to a community teaching hospital to determine its potential role in the treatment of COVID-19.

## 2. Experimental Section

### 2.1. Patients and Study Design

**Figure 1:**
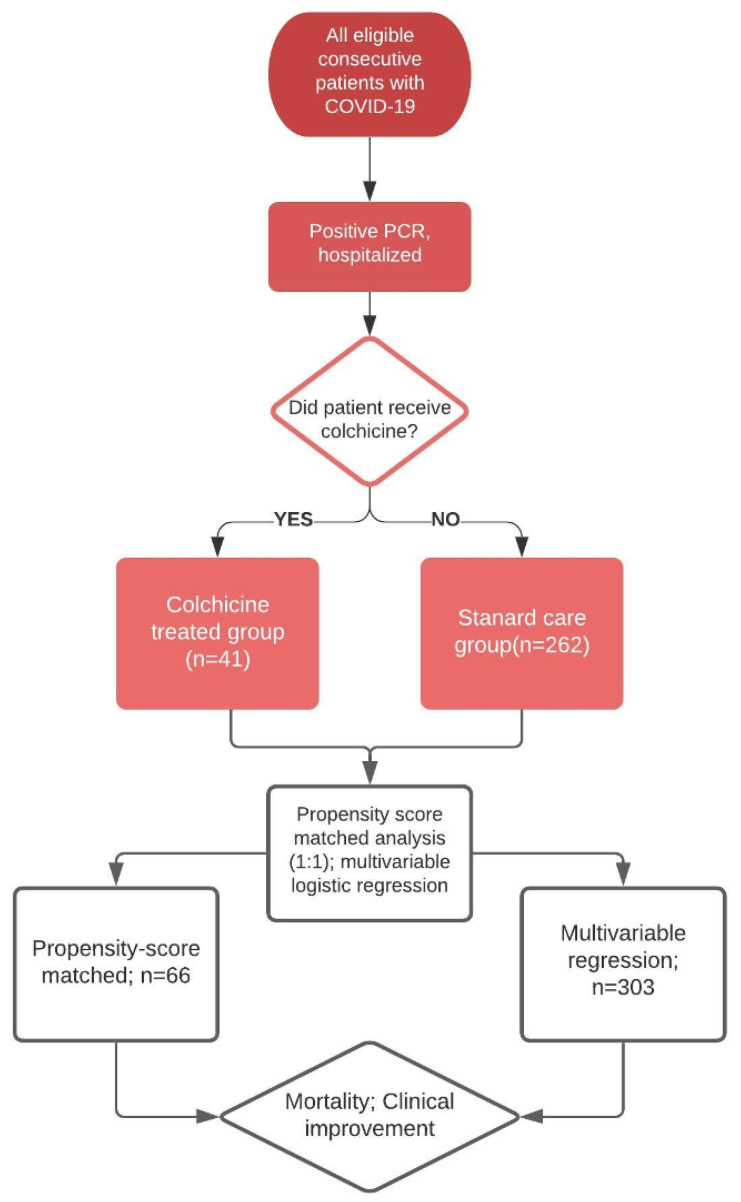
Overview of study data selection and analysis.

A single-center propensity score matched cohort study, including all consecutive severe COVID-19 patients with confirmed SARS-CoV-2 infection (positive PCR) admitted to a community teaching Hospital between 1 March 2020 and 30 May 2020, was performed. Patients were stratified according to the receipt of colchicine. The institution developed a COVID-19 patient care team and treatment guidelines, which were deployed at the start of the COVID-19 crisis at the medical center. The timing and choice of colchicine administration was dependent on the individual clinician; however, the general approach was to administer colchicine early before the progression of respiratory failure in patients who were given the medication. The dosage was based on the gout flare dosage and an assessment of ongoing COVID-19 clinical trials summarized in our recent review [7]. This open-label study did not have a predetermined number of patients or duration. The administration of other medications for the treatment was based on the institutional protocol and under the direction of the hospital COVID-19 team. During the study, hydroxychloroquine and azithromycin were prescribed in all patients unless the clinician felt there was a risk of toxicity (i.e., QT prolongation, cardiac risk). Remdesivir was administered only in the context of a clinical trial or under the expanded-use access program. Tociluzimab was prescribed on a case-by-case basis after careful patient assessment and evidence of cytokine storm based on the ferritin concentration > 300 ng/mL. Corticosteroids were not routinely prescribed nor recommended by the institutional guidelines at the time of this study since at the time, there were inadequate data to support the safety of their administration for COVID-19. Of note, as data emerged throughout the pandemic, the institutional guidelines were updated to reflect best practice based on available evidence. Both hydroxychloroquine and azithromycin were removed from the institutional guideline recommendations in early May, 2020. Figure 1 provides an overview of the study flow.

### 2.2. Study Assessments

We quantified key clinical events, laboratory data, hospital discharge, and reported adverse events, including those leading to discontinuation of treatment, serious adverse events, and death. To ascertain the level of comorbidity, we calculated the Charlson-Deyo comorbidity index for each patient [12].

In addition, we evaluated the modified Ordinal Scale for Clinical Improvement (OSCI), as recommended by the World Health Organization (WHO) R&D Blueprint Group at baseline and on days 7, 14, and 28 [13]. The OSCI was scored as follows: (0) no clinical or virologic evidence of infection, (1) no limitation of activities, (2) limitation of activities, (3) hospitalized, no oxygen therapy, (4) oxygen by mask or nasal prongs, (5) non-invasive ventilation or high flow oxygen, (6) intubation and mechanical ventilation, (7) ventilation plus additional organ support (i.e., pressors, renal replacement therapy, extracorporeal membrane oxygenation), or (8) death. At each assessment, the worst (i.e., highest ordinal) score was recorded.

The primary endpoint was defined as all-cause in-hospital death within the 28-day follow-up. The secondary endpoints were defined as a favorable change in OSCI on days 14 and 28 versus baseline, proportion of patients with a WHO OSCI score of <4 (indicating no need for supplemental oxygen on days 14 and 28, and proportion of patients discharged on day 28).

Baseline characteristics of the patients: demographics, clinical including drugs, adverse events, and laboratory values, including serum creatinine, lactate dehydrogenase (LDH), lactic acid, inflammatory markers including C-reactive protein (CRP) and ferritin were extracted from patients’ electronic medical records. All data were extracted from the electronic medical record, and at least two independent investigators adjudicated study data before analysis.

### 2.3. Institutional Review Board

The study was granted expedited approval and a waiver of consent by the Robert Wood Johnson University Hospital Somerset (IRB20-20) and Rutgers Biomedical and Health Sciences (PRO2020001113) Institutional Review Boards and conforms to the Strengthening the Reporting of Observational Studies in Epidemiology (STROBE) statement [14].

### 2.4. Statistical Analysis

To account for the treatment strategy, since it may be influenced by confounding indication (the tendency of clinicians to prescribe colchicine in patients perceived to have cytokine storm and worsening trajectory), propensity score matching was performed. Propensity scores were calculated using a multivariable logistic regression model where colchicine was the dependent variable. Covariates were selected a priori based on the likelihood that they would influence clinicians to prescribe colchicine and included in the propensity score. The variables included age, sex, body mass index (BMI), select baseline laboratory values (serum creatinine, LDH, lactic acid, ferritin, CRP, procalcitonin), baseline oxygen saturation on room air, receipt of tocilizumab, receipt of remdesivir, and comorbidity score. The comorbidity score includes important confounders related to the worse prognosis in COVID-19, including diabetes and chronic pulmonary disease among others. Individual diseases were not included into the propensity score since doing so would be redundant. We used the nearest-neighbor approach without replacement, and a caliper width of 0.2 controls was selected 1:1. Standardized mean biases were tested and visually inspected using a dot plot to ensure balance after propensity score matching between groups. In the event of missing data, multiple imputation was used to input predicted values. All data were summarized using descriptive statistics. Chi square test was used for categorical data and the Student’s t-test was used for continuous data. The odds ratio for the primary endpoint, all-cause in-hospital death within the 28 days, and corresponding 95% confidence intervals were calculated using logistic regression. Similarly, the odds ratio and 95% confidence intervals were calculated for all secondary endpoints. As a sensitivity analysis and due to concern for residual confounding, we performed a multivariable logistic regression using the entire dataset (unmatched) to evaluate the primary outcome of mortality. Confounders were selected for inclusion in the model based on biologic plausibility for association with the exposure (colchicine) and the outcome. A bivariate analysis was performed and only those variable with a *p* < 0.01 were further evaluated in multivariable logistic regression. Variables with a *p* < 0.05 were retained in the final model. All data were analyzed using SPSS version 26.0 (IBM Corporation, Armonk, NY, USA).

### 2.5. Role of the Funding Source

There was no funding received for the completion of this study.

## 3. Results

### 3.1. Evaluation of Outcomes

#### 3.1.1. Baseline Characteristics of the Patients

Table 1 shows baseline demographic, clinical, and laboratory characteristics of the patients. There were 303 patients included in the initial dataset of which 41 received colchicine for a clinical status consistent with cytokine storm. After propensity score matching, 33 patients were selected as control patients and matched with 33 patients who received colchicine.

**Figure 2:**
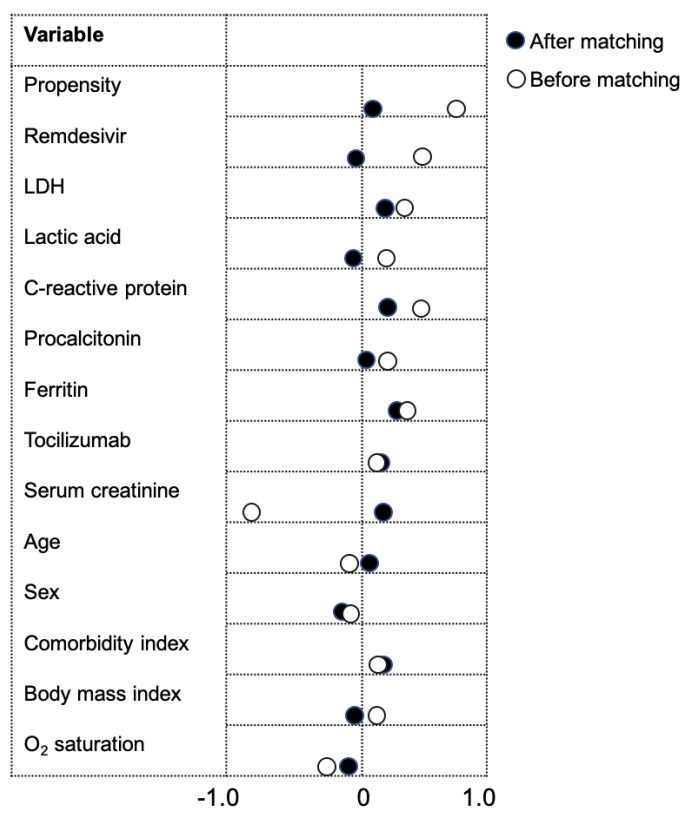
Standardized mean difference in covariates included in propensity score matching. The dot plot provides a graphical display of the standardized mean difference in covariates before and after propensity score matching. After matching the differences between groups were similar or improved. O_2_ saturation represents the value at baseline or hospital presentation.

Figure 2 illustrates the change in standardized mean difference between covariates included in the propensity score before and after matching. The median age of the total study population was 62.9 years (range: 26–97 years), and 34.8% (*n* = 23) were female. The most prevalent chronic comorbidities included hypertension (48.5%; *n* = 32), diabetes (21.2%; *n* = 14), obesity (45.5%; *n* = 30) and chronic pulmonary disease (13.6%; *n* = 9). The mean body mass index was 30.7 ± 7.4 kg/m^2^ and the comorbidity index score was 1.15 ± 1.7.

#### 3.1.2. Treatment with Colchicine

Of the 33 patients treated with colchicine included in the propensity matched cohort, 72.7% (*n* = 24) of the patients received a loading dose of 1.2 mg. The maintenance dose was 0.6 mg twice daily. Treatment was initiated within 72 h of hospital admission in 69.7% (*n* = 23) of patients.

#### 3.1.3. Concomitant Drugs Given

Patients also received concomitant hydroxychloroquine (54.5%; *n* = 18 versus 90.9%; *n* = 30), azithromycin (33.3%; *n* = 11 versus 78.8%; *n* = 26), tocilizumab (31.4%; *n* = 11 versus 33.3%; *n* = 11), and remdesivir (12.1%; *n* = 4 versus 12.1%; *n* = 4), when comparing colchicine versus standard of care, respectively.

#### 3.1.4. Clinical Improvement during Colchicine Treatment

**Figure 3:**
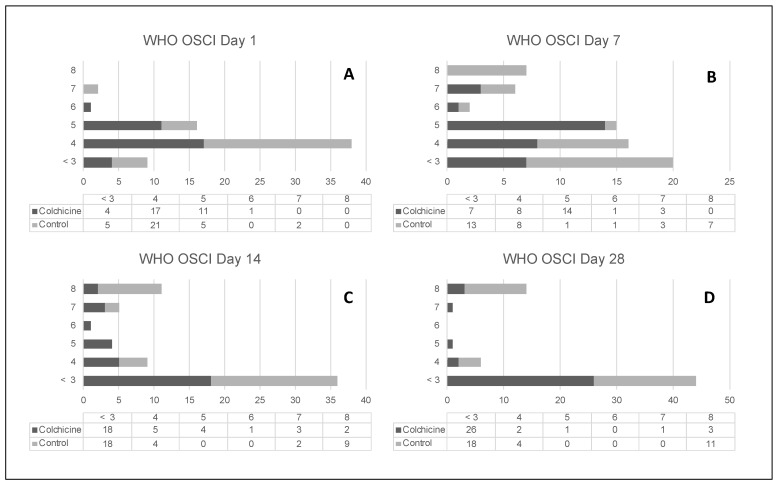
Figure (**A**–**D**) provide the distribution of WHO OSCI score at baseline and on days 7, 14, and 28. At baseline, only three patients required mechanical ventilation (WHO OSCI score above 6; colchicine = 1, control = 2). The last observation carried forward for each evaluation was used.

On day 14, both groups had a similar proportion of patients with a favorable change (improvement) in WHO OSCI score (57.6%; *n* = 19 versus 51.5%; *n* = 17; *p* = 0.621); colchicine versus standard of care, respectively. The proportion of patients with a WHO OSCI score < 4 (indicating no need for supplemental oxygen on day 14 was similar between groups (54.5%; *n* = 18 versus 54.5%; *n* = 18; *p* = 1.0); colchicine versus standard of care, respectively. On day 28, the receipt of colchicine was associated with a greater than 3-fold improvement in the WHO OSCI score and the WHO OSCI score < 4. Overall, significantly more patients were discharged home on day 28 in the colchicine group versus the standard of care group (90.9%; *n* = 30 versus 66.7%; *n* = 22 *p* = 0.023) (Table 2). Figure 3 provides a visual depiction of the changes in WHO OSCI from baseline.

#### 3.1.5. Mortality

There were three deaths (9.1%) in the colchicine group versus 11 deaths (33.3%) in the standard of care group (odds ratio, 0.20; 95% confidence interval, 0.05–0.80; *p* = 0.023) at the end of the 28 day follow-up period (Table 2). A sensitivity analysis using the entire dataset (unmatched) was performed. There were four deaths (9.8%) in the colchicine group versus 58 (22.1%) in the control group (unadjusted odds ratio, 0.38; 95% confidence interval, 0.13–1.11; *p* = 0.077). Colchicine was associated with a significant reduction in mortality after adjustment for age, comorbidity index, and c-reactive protein (odds ratio, 0.21; 95% confidence interval, 0.06–0.71; *p* = 0.012).

#### 3.1.6. Laboratory Data

Given the nature of this observational study, some laboratory data measures were not repeated in all study patients At baseline, markers of inflammation, including ferritin, CRP, and LDH, were elevated in the majority of patients (Table 1). The mean ferritin was 1298.0 ± 1061 ng/mL, mean CRP was 14.7 ± 8.8 mg/dL, and mean LDH was 397.4 ± 143.6 U/L. A repeat CRP after drug administration was available for 36% (*n* = 9) of patients in the colchicine group. In these patients, a significant reduction in mean CRP from baseline 14.8 ± 9.1 mg/dL versus 7.8 ± 6.0 mg/dL (*p* = 0.021) was observed.

## 4. Discussion

Currently, there are no FDA approved anti-inflammatory drugs to treat SARS-CoV-2 infection. To date, few data are available regarding the use of colchicine. There has been some debate as to whether colchicine will be beneficial in COVID-19 or potentially harmful [7,15,16,17,18,19]. However, a recent randomized controlled trial reported a significantly improved time-to-clinical deterioration in patients treated with colchicine versus standard medical treatment [16]. Scarsi and colleagues reported that in patients with severe COVID-19 pneumonia, colchicine treatment was associated with a lower risk of death versus standard care (hazard ratio, 0.15; 95% confidence interval, 0.06 to 0.37; *p* < 0.0001) [20]. Optimal timing of colchicine administration is yet to be defined [21,22], but our approach was early administration. Our study describes the clinical outcomes of a small cohort of patients who received colchicine versus a propensity score matched control group. The primary outcome, all-cause in hospital mortality within 28 days was significantly reduced in patients who received colchicine (*p* = 0.012). Colchicine was associated with a significant five-fold increase in hospital discharge by day 28. WHO OSCI score was improved by day 28 and more patients had a WHO OSCI score of <4. In addition, there was significant decrease in CRP in patients in the colchicine group compared to the control group (*p* = 0.021). The wide confidence intervals suggest that the study suffers from a type II error. Data from several ongoing randomized, controlled trials will soon provide more information regarding the safety and efficacy of colchicine for COVID-19, the outcomes observed in this cohort study are the best currently available data.

Colchicine has anti-inflammatory and anti-viral properties. Colchicine forms a complex with tubulin, which leads to its anti-inflammatory effects, including inhibition of neutrophil chemotaxis, adhesion, and mobilization, and disruption of superoxide production, inflammasome and tumor necrosis factor inhibition, as well as possessing anti-viral properties. The intracellular transport of viral particles in the host cell, including particle trafficking to assembly sites in later stages of the infection, is mediated by microtubules and associated proteins, and this is part of the mechanism by which colchicine is proposed to work against COVID-19 [8]. A reduction in viral load is associated with a reduction in secreted pro-inflammatory mediators. It has been suggested that colchicine’s inhibitory effect on the activation, destabilization, and degradation of inflammasomes was partly responsible for interrupting the cytokine storm in COVID-19 [7,18].

Older men, and those with preexisting hypertension and/or diabetes as well as obesity, were highly prevalent in this cohort and the pattern was similar to data reported by others. The most common comorbidities seen in this cohort were hypertension (48.5%), diabetes (21.2%), and obesity (45.5%). The pattern was similar to data reported in a recently published large case series of hospitalized patients with confirmed COVID-19 in the United States [23].

This study has several limitations. Unfortunately, the duration of colchicine therapy was not entirely uniform in our study. The lack of uniformity was likely related to limited evidence on the use of colchicine in COVID-19 and prescriber preference. Many patients received a 1.2 mg loading dose (~73%), but this was not consistent. In terms of safety, there were no adverse events reported in patients who received colchicine in this cohort study. The retrospective nature of this study made it difficult to identify adverse events. Considering that gastrointestinal effects are common with both colchicine and with COVID-19, clinicians may not have reported events because of attribution to the disease process. There was a difference in the proportion of patients receiving hydroxychloroquine and azithromycin in the colchicine group versus the control group. During the study period, our institution removed these drugs from the COVID-19 treatment protocol based on available evidence suggesting lack of effect and possible toxicity [2].

The study results are limited by the small cohort size, the relatively short duration of follow-up, the fact that it is an observational study, and hence, there is difficulty in capturing all relevant clinical outcomes, owing to the nature of the study. In addition, the data were collected from the electronic health record database. This precluded the level of detail possible with a manual medical record review. Finally, sequential markers of inflammation were not available in the majority of patients, making it difficult to appreciate the impact of colchicine treatment on inflammation in the setting of COVID-19. Nonetheless, our findings support the safety and efficacy of colchicine in the treatment of patients with COVID-19 infection. This therapy warrants additional evaluation in large prospective randomized controlled studies.

## 5. Conclusions

In this cohort study, treatment with colchicine was associated with a higher rate of discharge and was associated with a decrease in mortality in patients with severe COVID-19 by day 28. These observations warrant further investigation in large controlled clinical trials.

## Figures and Tables

**Table 1:** Patient demographic and clinical characteristics before and after propensity score matching.

	Unmatched Patients			Propensity-Score Matched Patients		
	Colchicine	Standard (*n* = 262)	*p*-Value	Colchicine (*n* = 33)	Standard	*p*-Value
	(*n* = 41)				(*n* = 33)	
Age (years, mean ± SD)	61.2 ± 13.0	63.0 ± 16.4	0.496	61.7 ± 11.2	64.1 ± 15.4	0.473
Female (*n*, %)	13 (31.7)	103 (29.3)	0.352	12 (36.4)	11 (33.3)	0.796
Race (*n*.%)			0.712			0.694
White	11 (26.8)	68 (26.0)		9 (27.3)	8 (24.2)	
Hispanic	20 (48.8)	140 (53.4)		19 (57.6)	18 (54.5)	
African-American	3 (7.3)	26 (9.9)		1 (3.0)	3 (9.1)	
Asian	5 (12.2)	17 (6.5)		3 (9.1)	4 (12.1)	
Other	2 (4.9)	11 (4.2)		1 (3.0)	0 (0)	
LDH (U/L, mean ± SD)	410.9 ± 154.3	358.1 ± 144.4	0.032	402.6 ± 131.3	392.2 ± 156.8	0.773
Lactic acid (mg/dL, mean ± SD)	2.1 ± 2.0	1.8 ± 0.88	0.267	1.7 ± 0.6	1.9 ± 1.0	0.359
CRP (mg/dL, mean ± SD)	15.0 ± 9.0	10.9 ± 6.6	0.007	14.9 ± 8.9	14.4 ± 8.8	0.835
Procalcitonin (ng/mL, mean ± SD)	2.3 ± 8.9	0.66 ± 3.2	0.247	0.4 ± 0.5	0.6 ± 1.4	0.475
Ferritin (ng/mL, mean ± SD)	1315.7 ± 991.3	990.8 ± 918.0	0.038	1314.5 ± 1085.3	1281.5 ± 1052.8	0.901
Serum creatinine (mg/dL, mean ± SD)	0.87 ± 0.34	1.2 ± 1.4	0.132	0.9 ± 0.4	0.9 ± 0.5	0.801
Oxygen saturation (%, ± SD)	88.8 ± 6.4	90.7 ± 8.3	0.166	88.6 ± 6.3	86.24± 11.3	0.329
Body mass index (kg/m^2^, mean ± SD)	30.4 ± 7.4	30.0 ± 7.1	0.741	30.7 ± 7.0	30.6 ± 8.0	0.932
Comorbidity index score (mean ± SD)	1.4 ± 1.9	1.2 ± 1.8	0.561	1.3 ± 1.9	0.97 ± 1.5	0.392
Hypertension (*n*, %)	21 (51.2)	139 (53.1)	0.827	20 (60.6)	12 (36.4)	0.049
Myocardial infarction (*n*, %)	5 (12.2)	23 (8.8)	0.559	4 (12.1)	2 (6.1)	0.672
Heart failure (*n*, %)	3 (7.3)	14 (5.3)	0.712	3 (9.1)	2 (6.1)	1
Cerebrovascular disease (*n*, %)	5 (12.2)	18 (6.9)	0.215	5 (15.2)	2 (6.1)	0.427
Dementia (*n*, %)	5 (12.2)	22 (8.4)	0.386	4 (12.1)	2 (6.1)	0.672
Chronic pulmonary disease (*n*, %)	7 (17.1)	30 (11.5)	0.307	5 (15.2)	4 (12.1)	1
Diabetes (*n*, %)	8 (19.5)	84 (32.1)	0.047	7 (21.2)	7 (21.2)	1
Obese (*n*, %)	19 (46.3)	124 (47.3)	0.906	16 (48.5)	14 (42.4)	0.395
Hydroxychloroquine (*n*, %)	18 (43.9)	228 (87.0)	<0.001	18 (54.5)	30 (90.9)	0.001
Azithromycin (*n*, %)	12 (31.7)	167 (63.7)	<0.001	11 (33.3)	26 (78.8)	>0.001
Remdesivir (*n*, %)	10 (24.4)	9 (3.4)	<0.001	4 (12.1)	4 (12.1)	1
Tocilizumab (*n*, %)	13 (31.7)	73 (27.9)	0.612	12 (36.4)	11 (33.3)	0.796
Colchicine early administration * (*n*, %)		-	-	23 (69.7)	-	-
Colchicine loading dose (*n*, %)		-		24 (72.7)	-	-
WHO OSCI Score (*n*, %) **						0.216
≤3				4 (12.1)	5 (15.2)	
4				17 (51.5)	21 (63.6)	
5				11 (33.3)	5 (15.2)	
6				1 (3.0)	0 (0)	
7				0 (0)	2 (6.1)	

* Defined as administration within 72 h of admission; ** World Health Organization Ordinal Scale for Clinical Improvement, WHO OSCI.

**Table 2:** Comparison of endpoints between groups.

	Colchicine (*n* = 33) (*n*, %)	Standard (*n* = 33) (*n*, %)	Odds Ratio (95% Confidence Interval)	*p*-Value
**Primary endpoint**				
All-cause mortality by day 28	3 (9.1)	11 (33.3)	0.20 (0.05–0.80)	0.023
**Secondary endpoints**				
Favorable change in WHO OSCI on day 14	19 (57.6)	17 (51.5)	1.28 (0.48–3.37)	0.621
WHO OSCI * score of <4 (indicating no need for supplemental oxygen on day 14	18 (54.5)	18 (54.5)	1.0 (0.38–2.64)	1.000
Favorable change in WHO OSCI on day 28	26 (78.8)	17 (51.5)	3.50 (1.19–10.28)	0.023
WHO OSCI score of <4 (indicating no need for supplemental oxygen on day 28	26 (78.8)	18 (54.5)	3.10 (1.05–9.11)	0.040
Patients discharged on day 28	30 (90.9)	22 (66.7)	5.0 (1.25–20.08)	0.023

* World Health Organization Ordinal Scale for Clinical Improvement, WHO OSCI.
